# Mitochondrial Supercomplex‐Associated Proximity Increases in Malignant Glioma Tissue

**DOI:** 10.1096/fj.202600937RR

**Published:** 2026-10-13

**Authors:** Minou Tsujishita, Haesoo Bae, Anwaar Ali, Shanmuganathan Balakrishnan, Amina Abulimiti, Nelofer Syed, Kambiz N. Alavian

**Affiliations:** ^1^ Department of Brain Sciences, Faculty of Medicine Imperial College London UK

## Abstract

In rapidly growing tumors, including gliomas, heterogeneous microenvironments with fluctuating oxygen and nutrient availability impose strong selective pressure for metabolic adaptation. For over a century, mitochondria have been recognized as central to the reprogramming of cancer metabolism. However, it remains unclear whether and how remodeling of oxidative phosphorylation (OXPHOS), including organization of electron transport chain (ETC) complexes, contributes to the proliferation of cancer cells. Recent reports suggest that mitochondrial supercomplexes (SCs), higher‐order assemblies of ETC complexes, are enriched in tumor cells and may support proliferation by increasing respiratory efficiency and stabilizing electron transfer. Here, we evaluated respiratory SC formation across glioma grades by quantifying ETC complex proximity within mitochondria in patient tissue using proximity ligation assay (PLA). We observed increased PLA signal between ETC complexes I and IV, consistent with increased respirasome assembly, that was associated with malignant glioma (Grades III and IV), and increased proximity between ETC Complexes III and IV that was most prominent in proliferating glioblastoma (Grade IV) cells. Although the precise functions of SCs remain debated, these increases in high‐grade glioma relative to Grade II and control tissue suggest that SC remodeling may be a feature of malignant glioma metabolic remodeling.

## Graphical abstract

Higher‐order organization of the respiratory chain increases with glioma grade. Complexes I, III2, and IV associate into supercomplexes (SC) within mitochondria, and SC‐associated proximity rises progressively from control tissue through Grades II and III to Grade IV glioma, consistent with remodeling of respiratory chain organization during malignant progression.

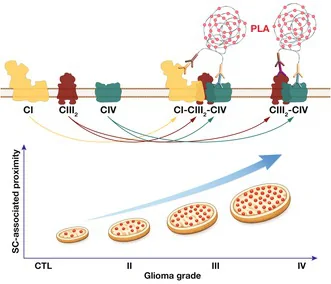

## 1 Introduction

Glioma is the most common primary tumor of the central nervous system. Representing the most prevalent form (> 50%) is glioblastoma multiforme (GBM), a highly aggressive malignant Grade IV astrocytoma characterized by a short median survival time of approximately 14 months and a 5‐year survival rate of 7.2% [1, 2]. Despite an improved understanding of the molecular mechanisms of GBM, survival rates remain poor under the current standard of care, including surgical resection, followed by radiotherapy and chemotherapy with temozolomide (TMZ) [3].

Tumor cells have long been believed to undergo metabolic reprogramming, primarily using glycolysis, even in the presence of oxygen, known as the Warburg Effect [4]. This shift from oxidative phosphorylation (OXPHOS) to glycolysis is considered advantageous for rapid ATP production, producing precursors for macromolecule synthesis, and protection of cancer cells from oxidative stress [5]. Recent evidence, however, has suggested dependence of many rapidly growing cancer cell types, including GBM, on both increased rates of glycolysis and OXPHOS [5, 6, 7].

The mitochondrial electron transport chain (ETC) is a series of protein complexes (CI, CII, CIII, CIV) embedded within the inner mitochondrial membrane [8], facilitating electron transfer and generating a proton gradient to drive ATP synthesis [9]. These mitochondrial complexes have been observed to form higher‐order organizations or supercomplexes (SCs), particularly in conditions where metabolic demand is high. The co‐existence of SCs and individual electron transport chain (ETC) complexes, as well as flexible organization of SCs, has been suggested to sustain cancer cells to support their energetic requirements and provide advantages for biogenesis. Recent cryo‐EM studies have shown that ETC complexes I, III, and IV are involved in SC formation, and three types of SCs (CI–CIII2–CIV (respirasome), CI–CIII2, and CIII2–CIV) have been observed in mammalian cells [10, 11, 12, 13]. Although the precise roles of the SC are yet to be elucidated, they have been proposed to increase the activity of the individual respiratory chain complexes by stabilizing them, enhancing OXPHOS by decreasing ROS production, or providing catalytic advantage [13, 14]. These functions might contribute to the energetic advantage required for the growth of cancer cells.

Here, we examined respiratory supercomplex organization across glioma grades at subcellular resolution, using proximity ligation assay (PLA) to quantify the local co‐occurrence of ETC complexes within mitochondria. We observed increased CI–CIV and CIII2–CIV proximity in high‐grade glioma patient tissues, including GBM, in comparison to low‐grade glioma and control tissues. These results are consistent with a shift towards elevated mitochondrial metabolism within high‐grade gliomas. Given the association between mitochondrial respiratory capacity and cellular proliferation, our results suggest that increased mitochondrial SC assembly in GBM cells may support or reflect their high proliferative state.

## 2 Results and Discussion

Mitochondrial respiratory SCs may enhance respiratory efficiency to sustain the high energetic demand of proliferation. To test whether SC‐associated proximity signals track with tumor grade, we quantified in situ ETC complex proximity by proximity ligation assay (PLA), a fluorescence‐based technique that detects close molecular interactions by amplifying signals from antibody‐bound targets within < 40 nm and compared proliferating and non‐proliferating cells across glioma grades and control tissue.

To quantify CI‐containing assemblies consistent with respirasomes, we measured PLA signal between NDUFB8 (complex I) and COX4 (complex IV) within heatshock protein 60 (HSP60)‐positive mitochondria. Specificity and background were assessed using IgG‐based negative controls (Figure S1A). Antibody specificity in FFPE tissue is shown in Figure S1B, and blue‐native PAGE resolving individual complexes and higher‐order supercomplex‐containing bands in Figure S1C. Representative images are shown in Figure 1A. Cells were classified as proliferating or non‐proliferating based on proliferating cell nuclear antigen (PCNA) positivity (PCNA‐positive cells denoted by “+”). PLA puncta within the HSP60‐positive mitochondrial masks were quantified within perinuclear regions defined as within 5 μm of the nuclear boundary and were normalized to mitochondrial area. CI–CIV PLA signal was elevated across high‐grade glioma groups, including proliferating and non‐proliferating GBM and Grade III, compared with Grade II glioma and control tissue (Figure 1B). Group means were: G4+ mean 33.85 (*n* = 24 cases), G4 mean 30.83 (*n* = 24), G3+ mean 33.65 (*n* = 7), G3 mean 32.77 (*n* = 7), G2+ mean 15.49 (*n* = 21), G2 mean 16.30 (*n* = 23), and CTL mean 4.04 (*n* = 4). Ordinary one‐way ANOVA followed by Tukey's multiple‐comparisons test identified significant increases for each high‐grade group versus both Grade II groups and versus CTL (adjusted *p* range = 0.0003–0.0362, depending on the comparison). Surprisingly, no differences were detected within the same tumor grade. The “+” suffix denotes proliferating, PCNA‐positive, cells, while the corresponding group without the suffix denotes PCNA negative cells within the same tissue and tumor grade. The respirasome organization might be part of a broader metabolic adaptation of the tumor microenvironment rather than a feature of proliferation or cell‐cycle state. Cancer cells are known to communicate extensively with neighboring cells and to remodel their local microenvironment through metabolic and signaling interactions. Recent transcriptomic analyses also suggest that oncogenic signaling can induce growth‐promoting responses in neighboring non‐transformed cells [15]. It has also been shown that intermediate cellular states can exist between tumor and normal tissue [16]. The relatively similar PLA signal strength observed between proliferating (+) and non‐proliferating cells may therefore reflect shared metabolic adaptations across the tumor tissue.

**FIGURE 1:**
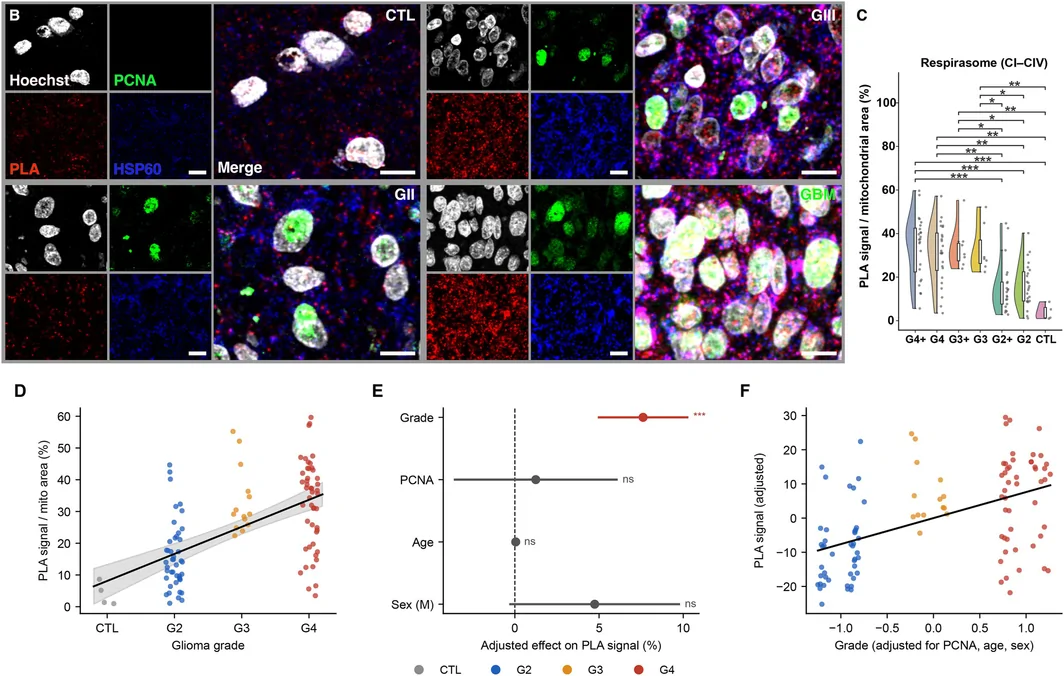
CI–CIV proximity signal (respirasome‐associated assemblies) is increased in high‐grade glioma tissue. (A) Representative confocal images from glioma and control tissue microarrays showing nuclei (Hoechst, white), proliferating cells (PCNA, green), mitochondria (HSP60, blue), and PLA puncta reporting CI–CIV proximity (red). Scale bars, 10 μm. (B) Quantification of CI–CIV PLA signal restricted to HSP60‐positive mitochondria and normalized to mitochondrial area within a perinuclear region of interest (within 5 μm of the nuclear boundary). Groups: G4+ (*n* = 24), G4 (*n* = 24), G3+ (*n* = 7), G3 (*n* = 7), G2+ (*n* = 21), G2 (*n* = 23), and CTL (*n* = 4). Each point represents one case. One‐way ANOVA with Tukey's multiple‐comparisons test. *p < 0.05; **p < 0.01; ***p < 0.001. (C–E) Multiple linear regression of CI–CIV PLA signal on tumor grade, PCNA positivity, age, and sex across tumor cases (106 observations from 57 cases). (C) CI–CIV PLA signal as a function of glioma grade, with the fitted regression line and 95% confidence band; each point represents one case, colored by group (CTL, gray; G2, blue; G3, orange; G4, red—Control cases are plotted in gray for reference but were not included in the regression model, which was fitted across tumor cases only). (D) Coefficient plot showing the adjusted effect (regression coefficient ±95% confidence interval) of each predictor; tumor grade was the only significant predictor (*β* = 7.61 per grade increment, ***p < 0.0001), whereas PCNA positivity, age, and sex were not significant (ns). The overall model was significant (*F*_4,101_ = 11.08, *p* < 0.0001; *R* ^2^ = 0.305, adjusted *R* ^2^ = 0.278). (E) Partial‐regression (added‐variable) plot for tumor grade, showing the grade–PLA relationship after adjustment for PCNA positivity, age, and sex. ns, not significant.

To explore whether patient demographics or proliferation state, in addition to tumor grade, influenced CI–CIV proximity, we performed multiple linear regression analysis across glioma grades, incorporating tumor grade, PCNA positivity, age, and sex as predictors of CI–CIV PLA signal (Figure 1C–E). The model was statistically significant (*F*_4,101_ = 11.08, *p* < 0.0001), explaining 30.5% of the variance (*R* ^2^ = 0.305, adjusted *R* ^2^ = 0.278). CI–CIV proximity was not affected by proliferative status, age, or sex (PCNA positivity [*p* = 0.61], age [*p* = 0.52], and sex [*p* = 0.06]), whereas tumor grade had a significant effect (*β* = 7.61 per grade increment, *p* < 0.0001) (Figure 1D). The grade‐associated increase in CI–CIV proximity persisted after adjustment for PCNA positivity, age, and sex (Figure 1E). These findings reveal that tumor grade, rather than proliferative state or demographic factors, is the primary determinant of CI–CIV proximity signal.

We also quantified proximity between UQCRC2 (complex III) and COX4 (complex IV) within HSP60‐positive mitochondria. Representative images and quantification are shown in Figure 2A,B. Ordinary one‐way ANOVA followed by Tukey's multiple‐comparisons test identified significant increases for G4+ versus G2+ (mean difference 9.548; adjusted *p* = 0.0178), G4+ versus G2 (mean difference 8.616; adjusted *p* = 0.0314), and G4+ versus CTL (mean difference 16.11; adjusted *p* = 0.0090). No differences were detected among the high‐grade groups (G4+, G4, G3+, and G3). Non‐proliferating GBM cells did not reach significance versus CTL after correction (G4 versus CTL, adjusted *p* = 0.0509). Because PLA reports molecular proximity rather than a defined stoichiometric assembly, these findings are most consistent with increased local co‐occurrence of complexes III and IV within mitochondria, compatible with enrichment of III‐IV‐containing assemblies in proliferating GBM cells, including both CIII2–CIV modules and CI–CIII2–CIV respirasomes.

**FIGURE 2:**
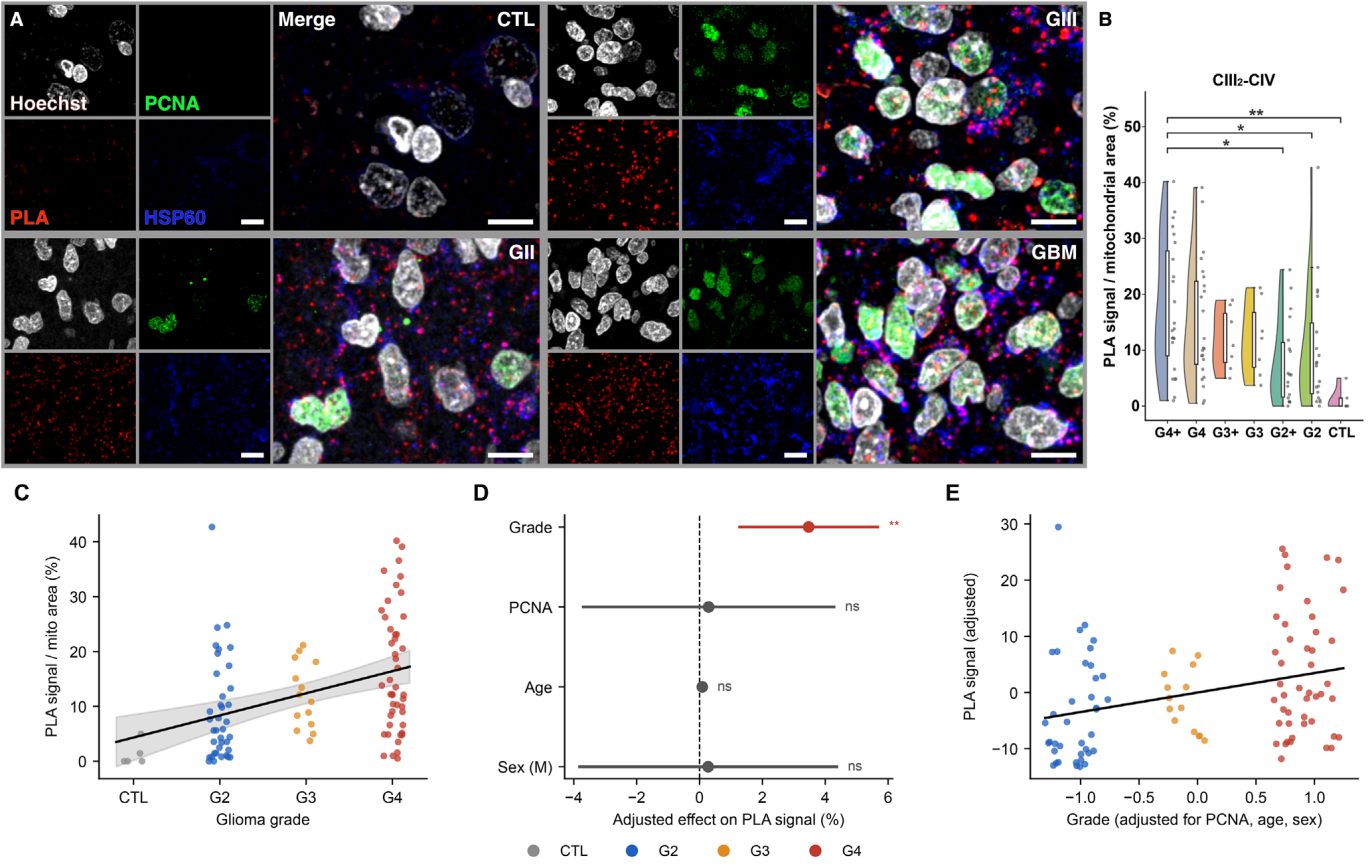
CIII2–CIV proximity signal (CIII–CIV‐containing SCs) is most prominent in proliferating GBM. (A) Representative confocal images showing nuclei (Hoechst, white), proliferating cells (PCNA, green), mitochondria (HSP60, blue), and PLA puncta reporting UQCRC2‐COX4 proximity (red). Scale bars, 10 μm. (B) Quantification of CIII2–CIV PLA signal restricted to HSP60‐positive mitochondria and normalized to mitochondrial area within the perinuclear region of interest (within 5 μm of the nuclear boundary). Groups: G4+ (*n* = 23), G4 (*n* = 23), G3+ (*n* = 7), G3 (*n* = 7), G2+ (*n* = 17), G2 (*n* = 21), and CTL (*n* = 5). Each point represents one case. One‐way ANOVA with Tukey's multiple‐comparisons test. *p < 0.05; **p < 0.01. (C–E) Multiple linear regression of CIII2–CIV PLA signal on tumor grade, PCNA positivity, age, and sex across tumor cases (98 observations from 57 cases). (C) CIII2–CIV PLA signal as a function of glioma grade, with the fitted regression line and 95% confidence band; each point represents one case, colored by group (CTL, gray; G2, blue; G3, orange; G4, red. Control cases are plotted in gray for reference but were not included in the regression model, which was fitted across tumor cases only). (D) Coefficient plot showing the adjusted effect (regression coefficient ±95% confidence interval) of each predictor; tumor grade was the only significant predictor (*β* = 3.47 per grade increment, **p = 0.0023), whereas PCNA positivity, age, and sex were not significant (ns). The overall model was significant (*F*_4,93_ = 3.32, *p* = 0.014; *R* ^2^ = 0.125, adjusted *R* ^2^ = 0.087). (E) Partial‐regression (added‐variable) plot for tumor grade, showing the grade–PLA relationship after adjustment for PCNA positivity, age, and sex. ns, not significant.

We also explored whether patient demographics or cell proliferation state contributed to CIII_2_–CIV proximity by performing multiple linear regression analysis across cancer cases, incorporating tumor grade, PCNA positivity, age, and sex as predictors of CIII_2_–CIV PLA signal (Figure 2C–E). The model was statistically significant (*F*_4,93_ = 3.32, *p* = 0.014), explaining 12.5% of the variance (*R* ^2^ = 0.125, adjusted *R* ^2^ = 0.087). CIII_2_–CIV proximity was not affected by proliferative status, age, or sex (PCNA positivity [*p* = 0.89], age [*p* = 0.18], and sex [*p* = 0.89]), whereas tumor grade had a significant effect (*β* = 3.47 per grade increment, *p* = 0.0023) (Figure 2D). The grade‐associated increase in CIII_2_–CIV proximity persisted after adjustment for PCNA positivity, age, and sex (Figure 2E). Similar to the CI–CIV proximity, these findings reveal that tumor grade is the main determinant of CIII_2_–CIV proximity, although the smaller proportion of variance is in keeping with the more modest grade‐associated increase observed for this pairing.

Regulation of the assembly of respiratory complexes within the mitochondrial inner membrane into higher order SCs has been suggested as a feature of metabolic remodeling across several physiological states and diseases. Although the precise functions of SCs are yet to be elucidated, protection against reactive oxygen species, regulation of individual ETC complexes, and preventing electron leakage have been suggested as potential functions [13, 14, 17]. Recent work has connected increased SC assembly with hypoxia adaptation and therapy resistance in several cancers [13, 18]. In glioblastoma, increased SC organization has also been linked to reduced mitochondrial reactive oxygen species and radio resistance [19].

Our data suggest that SC‐associated proximity increases with malignant grade and proliferative state in glioma tissue. CI–CIV PLA signal was increased across high‐grade glioma groups relative to Grade II and control tissue, indicating a high‐grade shift towards respirasome formation. CIII2–CIV PLA signal was also increased in proliferating glioblastoma cells compared with Grade II and controls. It is noteworthy that although the antibodies against UQCRC2–COX4 PLA have been used to detect the CIII2–CIV SCs, CIII2–CIV also coexist in respirasomes. Lack of a detectable increase in Grade III, therefore, might suggest that the CIII2‐CIV SC remains near baseline in the lower grades, while the proliferating GBM cells, the most biosynthetically demanding cells, utilize both SCs for growth. PLA is a measure of molecular proximity, not a direct measure of the stable, stoichiometrically defined supercomplex formation. An increased PLA signal therefore reflects increased local co‐occurrence of the relevant complexes rather than a direct quantification of assembled SCs. PLA has nonetheless been used as a proxy for supercomplex formation in mitochondrial studies, where the proximity of respiratory complex subunits, including CI–CIII and CI–CIV, has been assessed and interpreted alongside biochemical assays of supercomplex assembly such as blue‐native PAGE [20]. In that study, the same CI–CIV antibody pairing was benchmarked directly against blue‐native PAGE across multiple genetic and pharmacological models of respiratory chain disruption. Taken together, these observations indicate that the grade‐associated increase in PLA signal may reflect remodeling of respiratory chain organization towards greater supercomplex assembly in high‐grade glioma.

Our analysis was performed on well‐characterized, commercially sourced tissue microarrays, providing a standardized, uniformly processed cohort spanning the full range of glioma grades alongside matched control tissue. This allowed direct, side‐by‐side comparison of SC‐associated proximity across grades under identical fixation, staining, and imaging conditions. Detailed cohort characteristics, including age, sex, tumor grade, and the number of specimens analyzed, are provided (Table S1). Grading followed the WHO classification of central nervous system tumors as characterized by the supplier. Molecular subtyping and clinical follow‐up were outside the scope of the present study and represent a natural direction for future work.

Rapidly dividing tumor cells require mitochondrial function not only for ATP production but also to sustain redox balance and enable anabolic pathways required for proliferation, including pathways that depend on respiratory electron acceptors to support amino acid availability and nucleotide synthesis [21]. It has also been shown that ETC organization can shift between different structural states as cells adapt between oxidative and glycolytic metabolism [22, 23], suggesting that SC formation and remodeling may contribute to metabolic plasticity instead of merely accommodating a fixed increase in energetic demand. The increased SC formation in high‐grade glioma is consistent with the possibility that malignant progression depends on more stable electron transfer, limited electron leak, and enhanced redox homeostasis across fluctuating tumor microenvironmental constraints, including variability in oxygen and nutrient levels [18].

## 3 Materials and Methods

### 3.1 Immunohistochemistry and Proximity Ligation Assay

Human tissue microarrays were obtained from US Biomax (BS17016c, GL803d) and comprised formalin‐fixed, paraffin‐embedded (FFPE) tumor samples collected from patients diagnosed with GBM and varying grades of glioma (II‐III) (Table S1), as well as control samples obtained from normal adjacent tissue. All cases were confirmed by the manufacturer. The cohort comprised gliomas of mixed histology: Grade II tumors included astrocytoma, oligodendroglioma, oligoastrocytoma, and ependymoma; Grade III tumors were anaplastic astrocytoma; and Grade IV tumors were glioblastoma. Control cores consisted of adjacent normal brain tissue. No PCNA positive cells were observed in the control images. Cases were grouped by WHO grade for all analyses, and grade was modeled as an ordered predictor in the multiple linear regression. Tumor grade was assigned according to the 2007 WHO classification of central nervous system tumors. The tissue microarrays contained only a single Grade I specimen (pilocytic astrocytoma). We excluded this sample as an independent group, as statistical analysis could not be performed on a single specimen. Analyses compared proliferating and non‐proliferating cells within tumor and non‐tumor (control) tissue and did not distinguish between different cell types within the tissue. Tissue sections were 5‐μm thick, and the slides were pre‐baked at 60°C for 30 min.

Immunohistochemistry and proximity ligation assay were performed as described previously [20, 24, 25]. Briefly, slides were deparaffinized by 3 × 15‐min incubations in xylene and rehydrated in decreasing grades of ethanol. Heat‐induced epitope retrieval was performed using sodium citrate buffer (10 mM sodium citrate, 0.05% Tween‐20; pH 6.0). Following the antigen retrieval, the slides were kept in methanol, running water and PBS for 10 min each, followed by blocking with Duolink Blocking Solution (Sigma‐Aldrich) for 1 h at 37°C. The slides were subsequently incubated overnight at 4°C with primary antibody pairs, diluted in Duolink Antibody Diluent. For the CI–CIV proximity assay, rabbit anti‐NDUFB8 (1:200, CUSABIO) was paired with mouse anti‐COX4 (1:100, Invitrogen). For the CIII–CIV proximity assay, rabbit anti‐UQCRC2 (1:150, CUSABIO) was combined with mouse anti‐COX4 (1:100). PLA was performed using the Duolink In Situ kit according to the manufacturer's protocol with minor modifications. In brief, antibody‐labeled slides were incubated with oligonucleotide‐conjugated PLA probes (PLUS and MINUS), followed by ligation and rolling‐circle amplification to generate fluorescent PLA puncta. Wash steps were carried out using Duolink In Situ Wash Buffer A and Wash Buffer B.

Following PLA, the slides underwent a second round of immunostaining to label proliferating cells and mitochondria. Slides were blocked in PBS containing 10% goat serum and 0.05% Tween‐20 and incubated overnight at 4°C with mouse anti‐PCNA (1:100, Thermo Fisher) and rabbit anti‐HSP60 (1:100, GeneTex), followed by fluorophore‐conjugated secondary antibodies for 1 h at room temperature. Finally, the tissues were stained with Hoechst 33342 for 15 min at room temperature.

### 3.2 Imaging and Image Analysis

Tissues were imaged using the Leica STELLARIS 8 inverted confocal microscope with a 63× oil immersion objective. Images were captured as *z*‐stacks with 0.5‐μm step size. *Z*‐stacks were processed using maximum intensity projection in FIJI/ImageJ. PLA signals were overlaid on 2D projections and quantified within a mitochondrial mask generated by thresholding the HSP60 channel after background subtraction (rolling ball algorithm; radius, 50 pixels), following our published methods [24], in PCNA+ and PCNA‐ cells. PLA particles of 0.3 μm and above were counted within regions of interest (ROIs) defined as perinuclear zones (within 5 μm from the nucleus). Image analysis was automated using a custom‐made macro (Data S1). In brief, maximum‐intensity projections were processed with a Gaussian blur (sigma = 2) and background subtraction (rolling‐ball algorithm; radius, 50 pixels) to reduce noise and improve signal clarity. Mitochondrial and PLA masks were generated by applying the Huang auto‐threshold followed by a fixed raw intensity threshold (5000 to 65 535 for the HSP60 channel and 3833 to 65 535 for the PLA channel), verified visually against the raw data for every image. The PLA mask was binarized and separated by watershed segmentation before particle analysis. For each image, 1–14 regions of interest (ROIs) were manually defined around individual nuclei, and an expanded ROI was generated using a custom macro (Imperial College London) that enlarged the original ROI by 5 μm; the region between the original and expanded ROIs was defined as the perinuclear cytoplasmic region, within which mitochondrial and PLA signals were quantified. Because mitochondrial content, reflected by HSP60 signal, can itself vary with tumor grade, PLA signal was expressed as the proportion of the HSP60‐defined mitochondrial area within each ROI that was occupied by thresholder PLA signal, reported as a percentage, so that the reported values represent proximity signal per unit of mitochondrial area rather than absolute mitochondrial abundance. Image processing and analysis were performed in FIJI/ImageJ (Version 2.16.0/1.54p), using the custom semi‐automated macro (Data S1), in which regions of interest were defined manually around individual nuclei and segmentation thresholds were confirmed visually for each image, with all subsequent ring generation, masking, and quantification executed automatically. Between 1 and 14 cells were quantified per case (typically 4–7). For the CI–CIV proximity assay, the number of cells analyzed was 121 (G4+), 115 (G4), 37 (G3+), 37 (G3), 73 (G2+), 110 (G2), and 25 (CTL), giving a total of 518 cells. For the CIII2–CIV assay, the number of cells analyzed was 139 (G4+), 149 (G4), 47 (G3+), 48 (G3), 64 (G2+), 131 (G2), and 20 (CTL), giving a total of 598 cells.

### 3.3 Cell Culture and Native PAGE

Patient‐derived primary GBM cells were cultured in DMEM/F‐12 supplemented with 10% fetal bovine serum, 1% penicillin/streptomycin, and 0.1 μg/mL amphotericin B, at 37°C in 5% CO_2_. To confirm antibody specificity, mitochondria were isolated by differential centrifugation and native PAGE was performed as described previously [26, 27], with minor modifications. Cells were homogenized in ice‐cold mannitol/sucrose buffer containing protease inhibitors; the homogenate was cleared at 1000× *g* (10 min, 4°C) and mitochondria pelleted at 13 000× *g* (10 min, 4°C). Mitochondria were solubilized in extraction buffer (50 mM NaCl, 50 mM imidazole, 2 mM 6‐aminohexanoic acid, 1 mM EDTA) with digitonin (6 μg per μg protein) for 20 min on ice and cleared at 17 000× *g* (15 min, 4°C). Samples were resolved on hand‐cast 3%–8% gradient gels using imidazole anode buffer (25 mM imidazole, pH 7.5) and tricine/imidazole cathode buffer (50 mM tricine, 7.5 mM imidazole, pH 7.5), transferred onto PVDF membranes, and immunoblotted with antibodies against NDUFB8 (1:1000; CUSABIO, CSB‐PA015655LA01HU), UQCRC2 (1:1000; CUSABIO, CSB‐PA025668ESR1HU), and COX IV (1:1000; Invitrogen, MA5‐31470), followed by HRP‐conjugated secondary antibodies (1:5000; Cell Signaling, 7074S and 7076S).

### 3.4 Statistical Analysis

Data were analyzed using GraphPad prism and are presented as mean ± SEM. Statistical analysis of PLA data was carried out using one‐way ANOVA followed by Tukey's post hoc multiple comparisons test. Multiple linear regression analysis, modeling CI–CIV or CIII2–CIV PLA signal as a function of tumor grade, PCNA positivity, age, and sex across malignant cases, was performed in Python (using the NumPy, pandas, and SciPy libraries); model significance (F‐statistic), variance explained (*R* ^2^ and adjusted *R* ^2^), and per‐predictor coefficients with *p* values are reported in the text and figure legends. Each case contributed up to two observations, corresponding to PCNA‐positive and PCNA‐negative cells within the same specimen. The reported model n refers to the number of observations (106 for CI–CIV and 98 for CIII2–CIV) drawn from 57 tumor tissues. Proliferative status was therefore a within‐case factor and was entered as an explicit predictor in the model, while tumor grade was a between‐case factor; in the grouped comparisons, each case contributes a single value to any one group. All biological replicates and statistical significance are reported in the text and figure legends.

## Author Contributions

Minou Tsujishita and Kambiz N. Alavian designed the experiments; Minou Tsujishita and Haesoo Bae performed the experiments and analyzed the data; Anwaar Ali, Shanmuganathan Balakrishnan, and Amina Abulimiti contributed to data interpretation and discussion; Nelofer Syed provided the patient derived cell line. Kambiz N. Alavian conceived, designed, and supervized the study, performed data quality control, and wrote the manuscript. All authors contributed important intellectual content, participated in writing, and read and approved the final version of the manuscript.

## Funding

The authors have nothing to report.

## Ethics Statement

The tissue microarrays used in this study were commercially sourced (US Biomax) from anonymized, de‐identified human specimens under the supplier's ethical approvals, and their use did not require additional institutional review.

## Conflicts of Interest

The authors declare no conflicts of interest.

## Supporting information


**Figure S1:** Validation of antibody specificity and supercomplex detection by proximity ligation assay (PLA). (A) PLA specificity controls in glioma tissue; the no‐primary‐antib control (Hoechst, PLA), the CI‐CIV and CIII2‐CIV PLA conditions (Hoechst, PLA), and the IgG‐IgG negative control (Hoechst, PLA, IgG, IgG). Minimal punctate signal in the IgG‐IgG and no‐primary controls confirms low non‐specific background. (B) Mitochondrial co‐localization of the individual ETC complex antibodies. Representative confocal images of glioma tissue stained for the mitochondrial marker HSP60 (blue) together with UQCRC2 (complex III), COX4 (complex IV), and NDUFB8 (complex I) (red), with the merged channel shown at right. Each complex antibody co‐localizes with HSP60‐positive mitochondria, confirming that the antibodies recognize their targets within the mitochondrial compartment in formalin‐fixed, paraffin‐embedded tissue. Scale bars, 10 Î¼m. (C) Blue‐native PAGE and immunoblot analysis of mitochondria isolated from patient‐derived primary glioblastoma cells, probed with NDUFB8, UQCRC2, and COX4 in three independent electrophoretic runs, resolving individual complexes and higher‐order supercomplex‐containing bands (CI‐, CIII2‐, and CIV‐containing assemblies, including CIII2‐CIV). Molecular‐weight markers (kDa) are indicated.

**Table S1:** Demographic and clinical characteristics of human brain cases, including age, sex, and CNS WHO grade for control brain tissue, Grade II‐III glioma, and Grade IV glioblastoma cases.

**Data S1:** Supporting Information.

## Data Availability

All data supporting the conclusions of this study are presented in the paper and its Supporting Information. Source data are available from the corresponding author upon request.
