# Supplementary material for: Mitochondrial Supercomplex‐Associated Proximity Increases in Malignant Glioma Tissue
Source: FASEB J. 2026 Oct 13;40(20):e72363. doi: 10.1096/fj.202600937RR (PMC13660765; doi:10.1096/fj.202600937RR)
Supplement: Supplementary file 1 [file FSB2-40-e72363-s001.pdf]

Group	Case No.	Age	Sex	Grade	Diagnosis
G4	23	43	F	4	Glioblastoma
G4	24	52	F	4	Glioblastoma
G4	25	58	M	4	Glioblastoma
G4	26	18	F	4	Glioblastoma
G4	28	59	F	4	Glioblastoma
G4	31	21	M	4	Glioblastoma
G4	32	45	F	4	Glioblastoma
G4	34	22	M	4	Glioblastoma
G4	35	1	M	4	Glioblastoma
G4	36	59	M	4	Glioblastoma
G4	37	58	M	4	Glioblastoma
G4	39	59	F	4	Glioblastoma
G4	40	42	M	4	Glioblastoma
G4	41	46	F	4	Glioblastoma
G4	43	48	F	4	Glioblastoma
G4	44	51	M	4	Glioblastoma
G4	45	61	F	4	Glioblastoma
G4	46	65	M	4	Glioblastoma
G4	47	63	F	4	Glioblastoma
G4	48	8	F	4	Glioblastoma
G4	49	9	M	4	Glioblastoma
G4	50	23	M	4	Glioblastoma
G4	51	39	M	4	Glioblastoma
G4	52	59	M	4	Glioblastoma
G4	53	17	M	4	Glioblastoma
G4	54	58	F	4	Glioblastoma
G4	55	42	M	4	Glioblastoma
G3	14	19	F	3	Anaplastic astrocytoma
G3	15	51	M	3	Anaplastic astrocytoma
G3	17	50	F	3	Anaplastic astrocytoma
G3	18	41	M	3	Anaplastic astrocytoma
G3	19	53	M	3	Anaplastic astrocytoma
G3	21	44	F	3	Anaplastic astrocytoma
G3	22	40	F	3	Anaplastic astrocytoma
G2	2	41	F	2	Astrocytoma
G2	4	53	M	2	Astrocytoma
G2	5	33	F	2	Astrocytoma
G2	6	39	F	2	Astrocytoma
G2	7	30	M	2	Astrocytoma
G2	8	43	F	2	Oligodendroglioma
G2	10	46	M	2	Astrocytoma
G2	12	25	M	2	Astrocytoma
G2	13	36	M	2	Astrocytoma
G2	16	36	F	2	Astrocytoma
G2	20	52	M	2	Astrocytoma
G2	27	25	M	2	Astrocytoma
G2	56	26	F	2	Oligoastrocytoma
G2	57	58	F	2	Oligoastrocytoma
G2	58	45	M	2	Oligoastrocytoma
G2	59	39	M	2	Oligoastrocytoma
G2	60	30	F	2	Oligodendroglioma
G2	61	39	M	2	Oligodendroglioma
G2	62	41	M	2	Oligodendroglioma
G2	63	54	M	2	Oligodendroglioma
G2	65	42	M	2	Oligodendroglioma
G2	66	48	M	2	Oligodendroglioma
G2	67	46	F	2	Oligodendroglioma
G2	68	44	F	2	Oligodendroglioma
G2	72	46	M	2	Ependymoma
G2	74	4	F	2	Ependymoma
CTL	76	30	F	CTL	Adjacent normal brain tissue
CTL	77	49	F	CTL	Adjacent normal brain tissue
CTL	78	30	M	CTL	Adjacent normal brain tissue
CTL	79	39	M	CTL	Adjacent normal brain tissue
CTL	80	62	M	CTL	Adjacent normal brain tissue

Supplementary Table 1. Demographic and clinical characteristics of human brain cases, including age, sex, and CNS WHO grade for control brain tissue, grade II-III glioma, and grade IV glioblastoma cases.
