# Supplementary material for: Mitochondrial Supercomplex‐Associated Proximity Increases in Malignant Glioma Tissue
Source: FASEB J. 2026 Oct 13;40(20):e72363. doi: 10.1096/fj.202600937RR (PMC13660765; doi:10.1096/fj.202600937RR)
Supplement: Supplementary file 2 [file FSB2-40-e72363-s003.docx]

**Supplementary Data File 1**

run("Set Measurements...", "area mean min area_fraction limit display redirect=None decimal=3");

//initialise

fn=getTitle();

roiManager("reset");

run("Clear Results");

run("Select None");

getDimensions(ImageWidth, ImageHeight, ImageChannels, ImageSlices, ImageFrames);

//project and filter

run("Z Project...", "projection=[Max Intensity]");

run("Gaussian Blur...", "sigma=2");

run("Subtract Background...", "rolling=50");

//hide channels 3 and 4

Stack.setActiveChannels("1100");

//draw rois

setTool("freehand");

waitForUser("Draw ROIS and add to roimanager (t)");

//create rings

n=roiManager("count");

for (roi=0;roi<n;roi++){

roiManager("select", roi);

run("Enlarge...", "enlarge=5");

roiManager("Add");

}

for (roi=0;roi<n;roi++){

roiManager("Select", newArray(roi,n+roi));

roiManager("XOR");

roiManager("Add");

}

roiManager("Select", Array.getSequence(n*2));

roiManager("delete");

for (roi=0;roi<n;roi++){

roiManager("select", roi);

roiManager("rename", "Ring "+roi+1);

}

//measure channel 4

selectWindow("MAX_"+fn);

run("Select None");

run("Duplicate...", "title=["+fn+" Mitohondria] duplicate channels=4");

setAutoThreshold("Huang dark");

run("Threshold...");

setThreshold(5000, 65535, "raw");

waitForUser("Check threshold for Mitochondria");

roiManager("deselect");

roiManager("measure");

roiManager("deselect");

run("Create Selection");

roiManager("Add");

close();

//measure channel 3

selectWindow("MAX_"+fn);

run("Select None");

run("Duplicate...", "title=["+fn+" PLA] duplicate channels=3");

setAutoThreshold("Huang dark");

run("Threshold...");

setThreshold(3833, 65535, "raw");

waitForUser("Check threshold for PLA");

setOption("BlackBackground", false);

run("Convert to Mask");

run("Watershed");

for (roi=0;roi<n;roi++){

roiManager("select", roi);

run("Analyze Particles...", "size=0.3-Infinity summarize");

}

//create coloc rings

for (roi=0;roi<n;roi++){

roiManager("Select", newArray(roi,n));

roiManager("AND");

roiManager("Add");

}

roiManager("deselect");

roiManager("Select", n);

roiManager("delete");

//measure coloc rings

for (roi=0;roi<n;roi++){

roiManager("select", roi+n);

roiManager("rename", "Coloc ring " +roi+1);

run("Analyze Particles...", "size=0.3-Infinity summarize");

}

close();

IJ.renameResults("Summary",fn+" PLA Count");

IJ.renameResults("Results",fn+fn+" Mito area");

selectWindow("MAX_"+fn);

Stack.setActiveChannels("1100");

roiManager("Show All");
